# Comparison of cardiac output and cardiac index values measured by critical care echocardiography with the values measured by pulse index continuous cardiac output (PiCCO) in the pediatric intensive care unit:a preliminary study

**DOI:** 10.1186/s13052-020-0803-y

**Published:** 2020-04-16

**Authors:** Nagehan Aslan, Dincer Yildizdas, Ozden Ozgur Horoz, Yasemin Coban, Fadli Demir, Sevcan Erdem, Yasar Sertdemir

**Affiliations:** 1 Department of Pediatrics, Division of Pediatric Intensive Care, Çukurova University Faculty of Medicine, Adana, Turkey; 2 Department of Pediatrics, Division of Pediatric Cardiology, Çukurova University Faculty of Medicine, Adana, Turkey; 3 Department of Biostatistics, Çukurova University Faculty of Medicine, Adana, Turkey

**Keywords:** Cardiac output, Cardiac index, Critical care echocardiography, PiCCO, Pediatric intensive care unit

## Abstract

**Background:**

Planning optimal fluid and inotrope-vasopressor-inodilator therapy is essential in critically ill children. Pulse index Contour Cardiac Output (PiCCO) monitoring is an invasive, hemodynamic monitor that provides parameter measurements such as cardiac output (CO), cardiac index (CI). Use of ultrasonography and critical care echocardiography by the pediatric intensivists has increased in recent years. In the hands of an experienced pediatric intensivist, critical echocardiography can accurately measure both CO and CI. Our objective in this study is to compare the CO and CI values measured by pediatric intensivist using critical care echocardiography to the values measured by PiCCO monitor in critically ill pediatric patients.

**Methods:**

A prospective observational study from a tertiary university hospital PICU. A total of 15 patients who required advanced hemodynamic monitoring and applied PiCCO monitoring were included the study. The diagnosis of patients were septic shock, cardiogenic shock, acute respiratory distress syndrome, pulmonary edema. Forty nine echocardiographic measurements were performed and from 15 patients. All echocardiographic measurements were performed by a pediatric intensive care fellow experienced in cardiac ultrasound. The distance of left ventricle outflow tract (LVOT) in the parasternal long axis and LVOT-Velocity Time Integral (LVOT-VTI) measurement was performed in the apical five chamber image. Cardiac output_echocardiography (CO_echo) and CI_echocardiography (CI_echo) were calculated using these two measurements. PiCCO (PiCCO, Pulsion Medical Systems, Munich, Germany) monitoring was performed. Cardiac output (CO_picco) and CI (CI_picco) were simultaneously measured by PiCCO monitor and echocardiography. We performed a correlation analysis with this 49 echocardiographic measurements and PiCCO measurements.

**Results:**

We detected a strong positive correlation between CO_echo and CO_picco measurements (*p* < 0.001, *r* = 0.985) and a strong positive correlation between CI_echo and CI_picco measurements (*p* < 0.001, *r* = 0.943).

**Conclusions:**

Our study results suggest that critical care echocardiography measurement of CO and CI performed by an experienced pediatric intensivist are comparable to PiCCO measurements. The critical care echocardiography measurement can be used to guide fluid and vasoactive-inotropic management of critically ill pediatric patients.

## Introduction

Pediatric patients in the pediatric intensive care units (PICU) are at higher risk for hemodynamic instability. Therefore, planning appropriate parenteral fluid and inotrope-vasopressor-inodilatory management is vitally important in critically ill pediatric patients [1]. Heart rate, mean arterial pressure, urine output, and other conventional methods monitoring techniques such as central venous pressure (CVP) are commonly used to assess the patient hemodynamic status. However, studies have reported that these parameters are subjective which led to the search for more advanced hemodynamic monitoring [2–4]. Recently, the importance of cardiac index (CI) in guiding fluid and inotropic management in septic shock was emphasized in the recent clinical practice parameters published in 2017 and 2020 which highlighted the significance of CI measurement in the PICU [5].

Over the past decades, CI monitoring techniques have changed from invasive to less invasive and non-invasive, respectively pulmonary artery catheterization, transpulmonary thermodilution and transthoracic doppler echocardiography [6]. Pulse index Contour Cardiac Output (PiCCO) monitor is a less invasive continuous cardiac output (CO) and hemodynamics monitor which works with transpulmonary thermodilution technology and does not require pulmonary artery catheterization. PiCCO use has increased in the PICU and it guides the pediatric intensivists in planning fluid and inotrope management of the patient by measuring continuous CO and CI, preload, systemic vascular resistance index by means of arterial thermodilution technique and arterial pulse contour analysis [7]. However, PiCCO monitor is an invasive and expensive technology which limits its utility and availability in all PICUs.

Echocardiography is becoming a standard of care in many intensive care units and more clinicians are learning how to perform bedside critical care echocardiography technique as more pediatric intensivist are becoming familiar with bedside ultrasonography in the PICU. The echocardiography type referred to as critical-care echocardiography has become a part of the routine evaluation of patients in the PICU by the pediatric intensivist [8]. This noninvasive technique allows the intensivist to measure and remeasure both CO and CI to guide patient management and ensure maintain hemodynamic stability to a critically ill patient.

These developments have brought to mind a variety of questions about hemodynamic monitoring. Intensivists are debating which monitoring technique (invasive or noninvasive) is more valuable for clinical practice [6]. The purpose of our study to detect the concordance between critical care echocardiographic CO and CI measurements performed by the pediatric intensivist with CO and CI measured by PiCCO in a tertiary PICU. Our goal is to emphasize the effectiveness of critical care echocardiography in the management of critically ill children when PiCCO monitoring is not feasible or available.

## Patients and methods

### Study design

We performed a prospective, observational study in PICU of a tertiary university hospital in south-eastern Turkey. All subjects who needed advanced hemodynamic monitoring and underwent PiCCO monitoring were included the study in 6 months study period. Echocardiographic measurements were performed in these patients, by one of the investigators (N.A.) who is experienced in critical care echocardiography and has performed more than 100 measurements. The study was approved by the Local Ethical Committee of the Medical Faculty of Çukurova University and written informed consent were obtained from parents.

### Study population

Fifteen patients with diagnosis of septic shock, cardiogenic shock, acute respiratory distress syndrome (ARDS), pulmonary edema who were hospitalized in our PICU and had PiCCO monitoring for unstable hemodynamics and uncertain volume status were included in the study. Demographic and clinical data including age, gender, body weight, underlying disease, Pediatric Index of Mortality (PIM2) score and Pediatric Risk of Mortality (PRISMIII) score were collected.

### Echocardiography technique

**Fig. 1:**
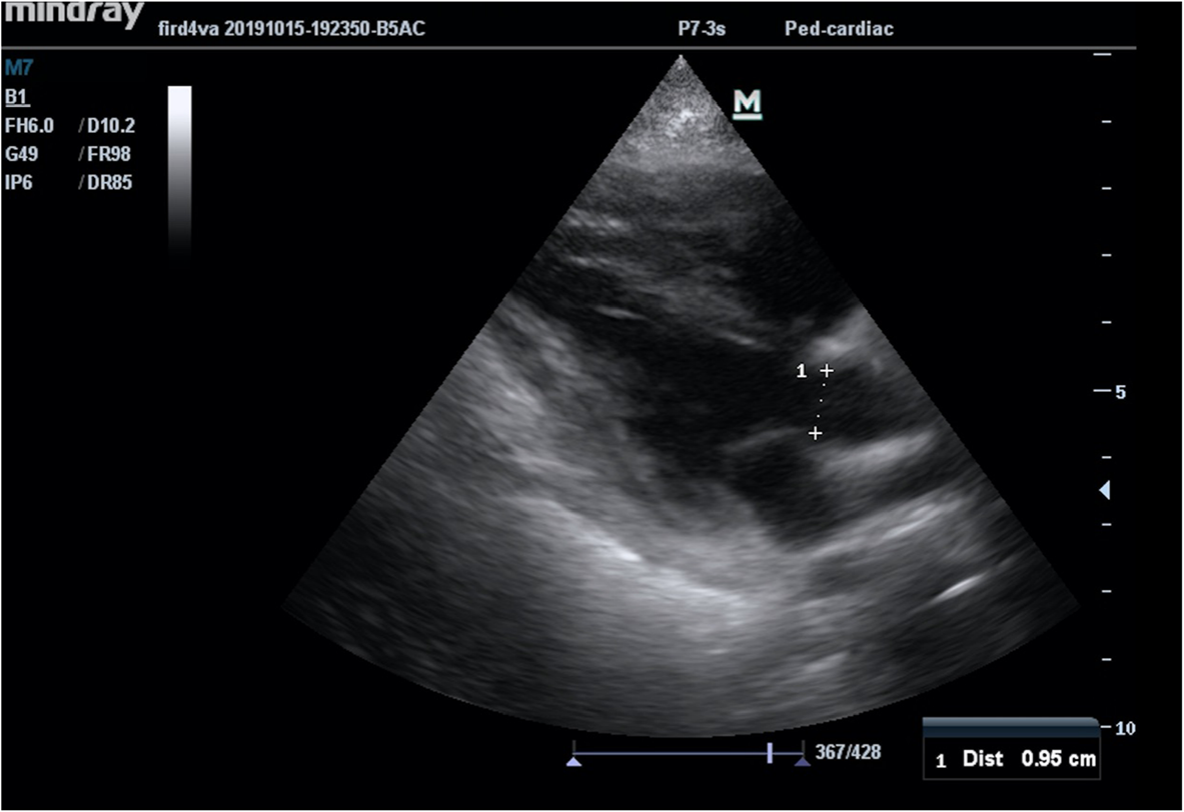
Measurement of the distance of LVOT in the parasternal longer axis

**Fig. 2:**
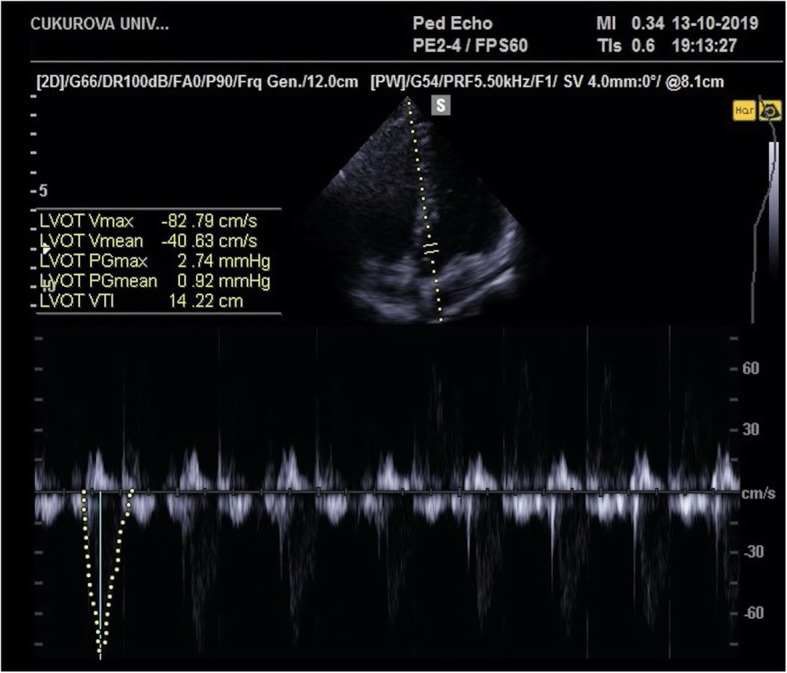
LVOT-VTI measurement in the apical five chamber image

Forty nine (49) echocardiographic measurements were performed simultaneously with the PiCCO measurements and recorded. Echocardiographic measurements were performed by a pediatric intensive care fellow (N.A.) who was trained in advanced ultrasound use and trained in critical care echocardiography by a pediatric cardiologists for 2 months. Measurements were done with the education ultrasound of our unit (Resona7, Mindray Bio-Medical Electronics Co., Ltd., China) with 6.0–7.0-MHz sector probe. First, the distance of left ventricle outflow tract (LVOT) in parasternal longer axis was measured (Fig. 1). In the apical 4 chamber image, LVOT-Velocity Time Integral (LVOT-VTI) measurement was performed by pulsed-wave Doppler on the aortic valve (Fig. 2) [9]. Stroke volume was calculated using these two measurements. Cardiac output_echocardiography (CO_echo) was calculated by stroke volume and heart rate multiplication (CO_echo = (Heart rate×LVOT-VTI × LVOT^2^ × 3.14)/4) [10]. Cardiac index_echocardiography (CI_echo) was computed by dividing the CO by the square meter of the patient. Every time, two measurements were done and their mean was recorded as CO_echo and CI_echo. All of the patients evaluated at least half an hour by N.A. for provide clear and certain results for echocardiographic measurements. The measurement method was reviewed with the pediatric cardiology department and it was approved. All measurements were approved by a pediatric cardiology specialist and a more experienced pediatric intensive care specialist who performed the same technique.

### PiCCO technique

PiCCO (PiCCO, Pulsion Medical Systems, Munich, Germany) monitoring which includes both the transpulmonary thermodilution and pulse contour technology was performed with a central venous catheter inserted into the internal jugular vein or subclavian vein of the patients and 3-french or 4-french thermodilution catheter (size selected according to the patient body weight) in which there is a heat sensor at the tip placed into the femoral artery. PiCCO system was calibrated with 10-ml cold saline every 8 h. The patients were normothermic during the PiCCO measurements. Cardiac output_picco (CO_picco) and cardiac index_picco (CI_picco) measured by PiCCO simultaneously with echocardiographic measurements were recorded by another physician who was blind to echocardiographic measurements.

### Statistical analysis

Data was analyzed using IBM SPSS Statistics Version 20.0 packet software. Categorical measurements were summarized as number and percent and the numeric data were summarized as mean and standard deviation (as median and minimum-maximum in some cases). For numeric measurements the assumption of normal distribution or not was tested with Kolmogorov-Smirnov/Shapiro Wilk test. We calculated the sample size as 10, which would find a correlation of at least 0.8 meaningful with 80% power. We performed our analysis on 49 simultaneous echocardiographic and PiCCO measurements from 15 patients. In order to assess the correlation between numeric measurements, Pearson Correlation Coefficient and the *p-*value were calculated. In all tests, the level of statistical significance was selected as 0.05.

## Results

The mean age of the patients was 93.2 ± 61.3 months and seven (46.6%) patients were females. The diagnoses of the patients were septic shock (8 patients), pulmonary ARDS (2 patients), cardiogenic shock due to scorpion sting (2 patients), pulmonary edema (3 patients). The demographic characteristics of the patients, CO_echo, the mean values of computed CI_echo, and simultaneously recorded PiCCO parameters were summarized in Table 1.

**Table 1:** The demographic characteristics of the patients, echocardiographic measurements and recorded PiCCO parameters

Characteristics, echocardiographic measurements and recorded PiCCO parameters	Mean ± SD	Median (min-max)
**Age (month)**	93.2 ± 61.3	101 (16–186)
**Body weight (kilograms)**	22.87 ± 12.75	20 (9.8–45)
**PIM 2 score**	47.1 ± 21.85	40.1 (19–95.1)
**PRISM III score**	22.4 ± 6.79	20 (15–41)
**CO_eko (L/min)**	4 ± 1.63	3.08 (1.6–7.59)
**CO_picco (L/min)**	3.7 ± 1.72	3.29 (1.65–8.5)
**CI_eko (L/min/m**^**2**^**)**	4.42 ± 1.02	4.45 (2.28–7.06)
**CI_picco (L/min/m**^**2**^**)**	4.57 ± 0.98	4.6 (2.47–7.32)

**Fig. 3:**
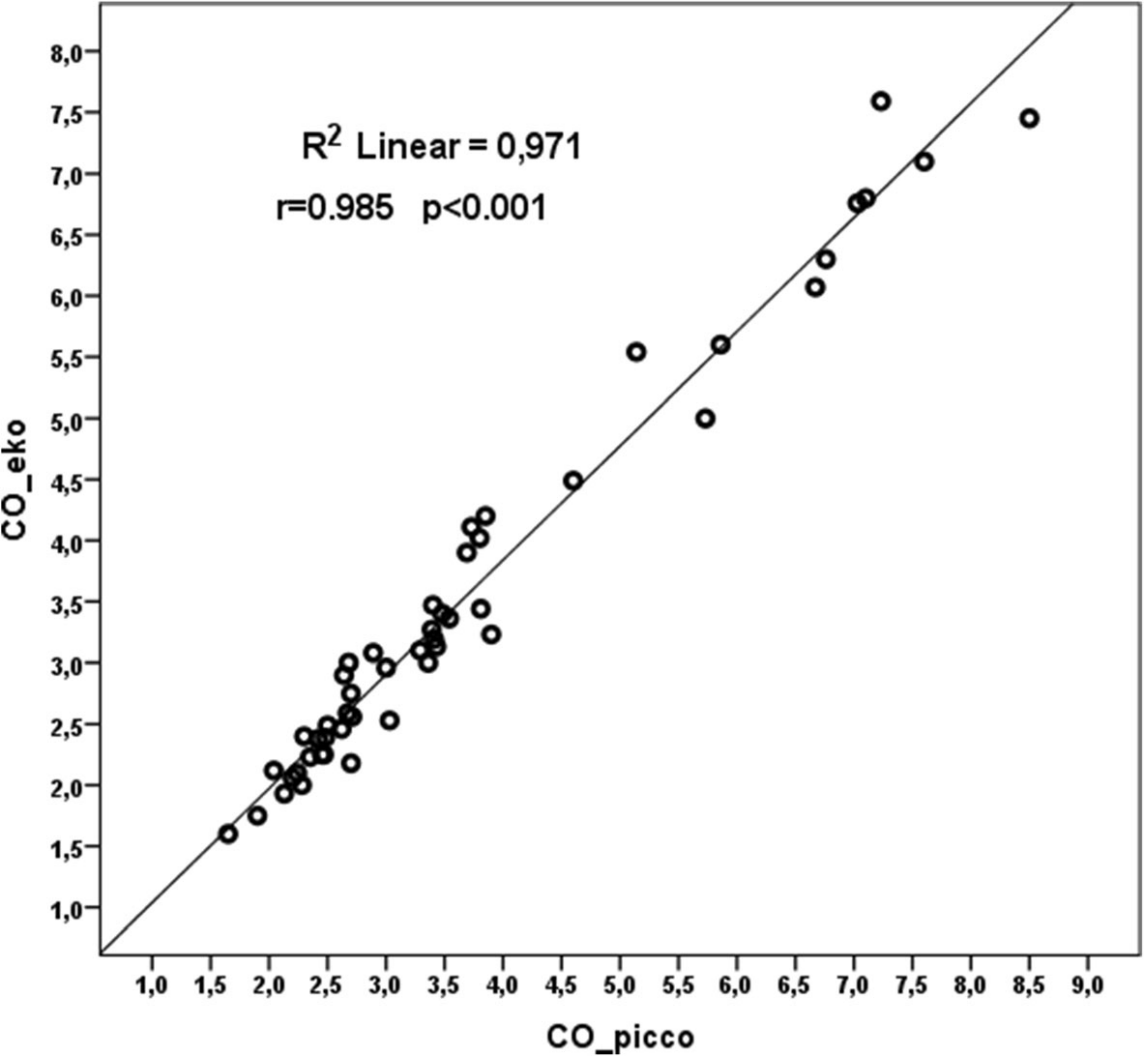
Correlation-regression curve of CO_echo and CO_picco measurements

**Fig. 4:**
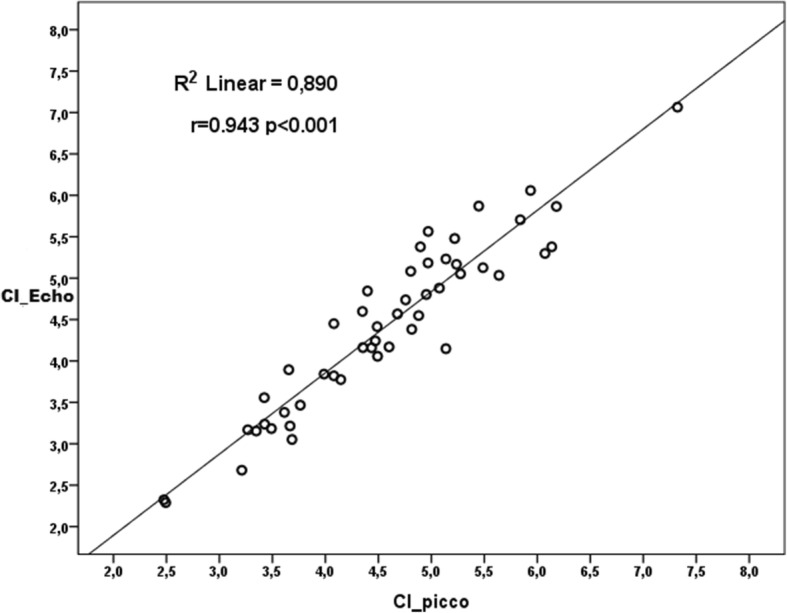
Correlation-regression curve of CI_echo and CI_picco measurements

**Fig. 5:**
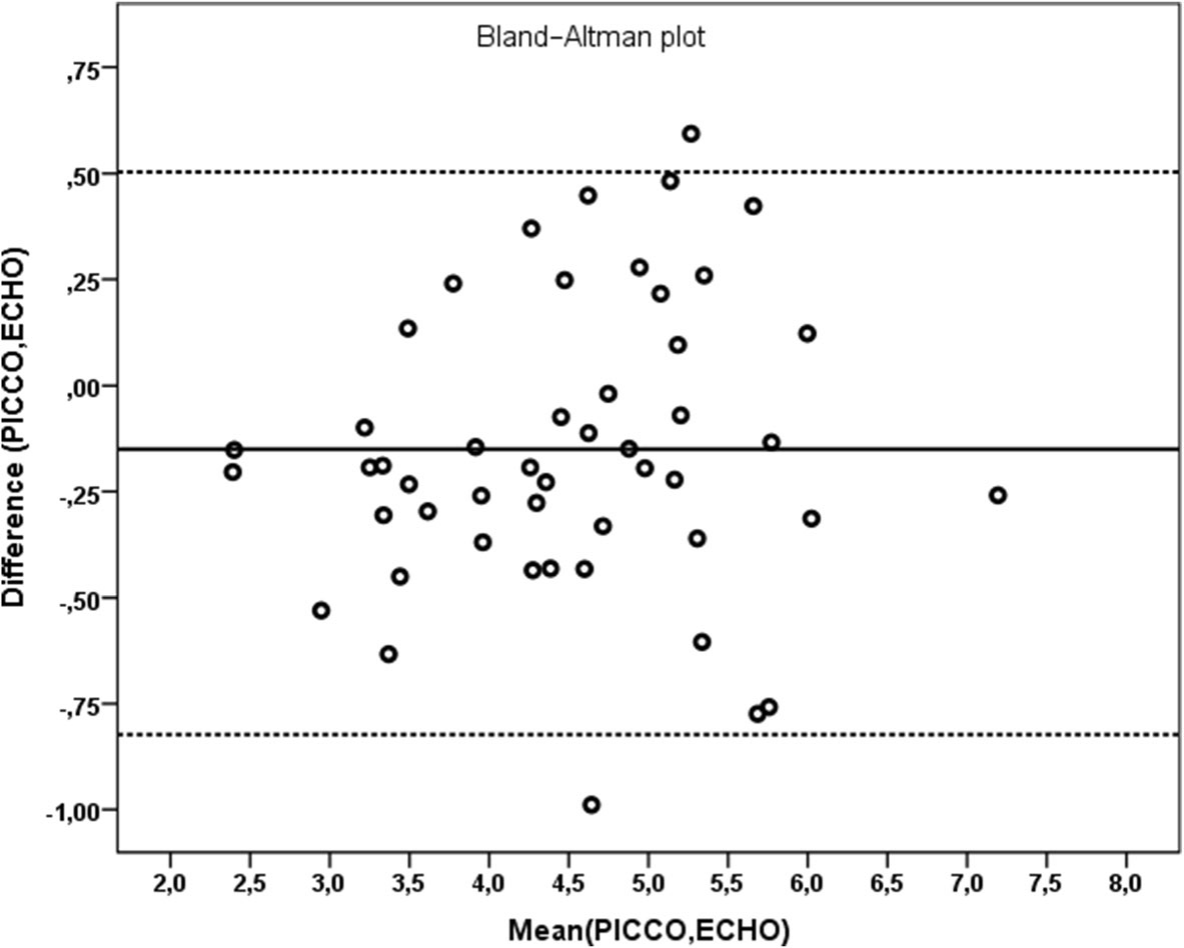
Bland-Altman graphic of mean difference between CI_echo and CI_picco measurements

Pearson correlation analysis showed a strong positive correlation between CO_echo and CO_picco measurements (*p* < 0.001, *r* = 0.985) and a strong positive correlation between CI_echo and CI_picco measurements (*p* < 0.001, *r* = 0.943) was detected (Figs. 3 and 4). The mean difference between CI_echo and CI_picco values was 0.15 and the standard error was 0.049 and it was detected that this difference is between 0.055 and 0.252 in 95%-confidence interval. You can see the Bland-Altman graphic of this difference in Fig. 5.

## Discussion

Planning optimal fluid and inotrope-vasopressor-inodilator management in critically ill pediatric patients is important. In children with shock, of any etiology, cardiac output measurement plays an important role in guiding fluid and inotrope treatment [11].

In the intensive care units, echocardiography, pulmonary artery catheterization and transpulmonary thermodilution methods are used to measure CO [12]. Pulmonary artery catheters offers monitoring several parameters including CO, CI, CVP, systemic vascular resistance index, and pulmonary capillary wedge pressure [12]. However, insertion of a pulmonary artery catheter into the right heart is an invasive procedure for the pediatric patients and can cause various cardiopulmonary complications [13]. Measuring CO with the thermodilution method, which is more invasive, in conjunction with pulmonary artery catheter is frequently used in adult patients and its use in the pediatric age group is limited [14–16]. However, it provides continuous measurement of CO, preload, myocardial contractility, afterload and pulmonary permeability when the pulse contour technology is added to transpulmonary thermodilution technology [17]. It has been shown that transpulmonary thermodilution technique correlates well with pulmonary artery catheter measurements [18]. Transthoracic doppler echocardiography can is a method for the assessment of CO and its effectivity has been proven in the estimation of cardiac output in critical patients [19, 20].

PiCCO is a less-invasive continuous CO and hemodynamic monitor which does not require pulmonary artery catheterization, and only needs a central venous catheter and femoral artery catheter [21]. Its working principle is based on transpulmonary thermodilution and pulse contour technology. While pulse contour analysis provides continuous CO and CI measurements, transpulmonary thermodilution is used to calibrate the system [1, 22]. During calibration, CO is calculated with the area under the curve. The measurement begins with standard thermodilution technique which enables monitoring the continuous CO with pulse contour analysis on the artery tracing. PiCCO technology is indicated in hemodynamically unstable patients and uncertain volume status. PİCCO is the first pulse contour device used for the CO measurement in clinical practice. It guides intensive care specialists in planning fluid and inotrope treatment to be applied by providing information on the patient preload and systemic vascular resistance [22]. Although some authors stated that PiCCO is the gold standard of care to identify the patient fluid status, most studies are in adults and pediatric data is limited [23]. In this study, we compared the CO and CI values measured by noninvasive critical care echocardiography with values measured by PiCCO in critically ill pediatric patients who were hospitalized in the PICU and required hemodynamic monitoring.

To the best of our knowledge, our study is one of few studies in the literature which compares CO measurements of transthoracic echocardiography and PiCCO in pediatric age group. Wurzer et al. [24] compared transthoracic echocardiography and PiCCO system in critically ill burned children. Their retrospective study results showed echocardiography derived estimates of CO may underestimate severity of the hyperdynamic state in severely burned children and they suggested that the PiCCO monitoring is a more objective way for observing cardiovascular and hyperdynamic states in critically ill pediatric patients [24]. Our results showed a strong and positive correlation between CO and CI levels measured by critical care echocardiography and PiCCO monitor. However, our study is a prospective study and the second difference is that echocardiographic measurements were performed by pediatric intensive care physicians. In our study, we specifically aimed to focus attention on the importance of bedside ultrasound and critical care echocardiography use by pediatric intensivists.

Gergely et al. [25] compared transpulmonary thermodilution, transthoracic echocardiography and conventional hemodynamic monitoring methods in neonates after open heart surgery in their study and they suggested that both transpulmonary thermodilution and transthoracic echocardiography may be used in the estimating volumetric preload parameters. The time course of transpulmonary thermodilution derived parameters may have clinical relevance in pediatric critical care practice [25]. Vignon et al. [11] compared transthoracic echocardiographic CO measurements performed with the CO measurements performed by transpulmonary thermodilution method in mechanically ventilated adult patients with septic shock and they reported that there is a mid-level concordance between the measurements. In a study which compared CO values measured by transthoracic echocardiography with the CO values measured with PiCCO in post cardiac arrest patients on therapeutic hypothermia. They found significant difference between the measurements of the patients in hypothermia group. Since echocardiographic measurement is not affected by body temperature, the difference was related to the thermal sensitivity of the PiCCO system [26]. A study comparing pulse contour and thermodilution methods with the CI measurements in pediatric cardiac surgery patients have noticed consistency between the two methods and a suggested that PiCCO system may be an optimal hemodynamic monitor in the pediatric cardiac surgery patients [18]. In a pediatric animal model, the stroke volume measured by echocardiography, thermodilution, and pulse contour were compared. The study found that echocardiographic measurements is consistent with transpulmonary thermodilution [17]. Our results showed a strong positive correlation between CO_echo and CO_picco measurements and a strong positive correlation between CI_echo and CI_picco measurements. The present study stands out in terms of being the first prospective study which suggests a noninvasive method for CI measurement instead of PiCCO in the pediatric literature. The most important aspect of our study is that the CO and CI measurements with critical care echocardiography were done by the pediatric intensivists.

This study had some limitations. Our sample size is small from a one medical center. The patient population was limited due to the fact that PiCCO is an invasive monitoring technique which can be applied in a selected patient group. We believe that further studies with larger patient groups will contribute to the literature. We are planning to continue working on increasing the number of patients and echocardiographic measurements. In order to minimize the technical differences which is one of the limitations of this study that could be originated fro.m the performer of the echocardiography, measurements were made by a pediatric intensive care fellow. This fellow was coordinated by a more experienced pediatric intensive care specialist. The measuring technique confirmed by the pediatric cardiologists and each measurement were made twice by N.A. and their averages were recorded. Additionally training and experience of the investigator about critical care echocardiography is very important for claim this correlation between the PiCCO and echocardiography mesurements.

## Conclusion

Due to various complications of invasive techniques, less invasive methods are preferred in intensive care units for hemodynamic monitoring. In recent years bedside ultrasound use has a rising trend and became more popular in PICU. Use of ultrasound especially critical care echocardiography has led to great advances in rapid, repetitive evaluation and intervention of critically ill pediatric patient. Based upon the results of this study, it is suggested that echocardiographic CO and CI measurements performed by an experienced pediatric intensive care and pediatric cardiology team may be as valuable as invasive PiCCO monitoring measurements in the planning of the treatment of critically ill pediatric patients.

## Data Availability

Please contact author for data requests.
